# Evaluating telehealth lifestyle therapy versus telehealth psychotherapy for reducing depression in adults with COVID-19 related distress: the curbing anxiety and depression using lifestyle medicine (CALM) randomised non-inferiority trial protocol

**DOI:** 10.1186/s12888-022-03840-3

**Published:** 2022-03-27

**Authors:** Lauren M. Young, Steve Moylan, Tayla John, Megan Turner, Rachelle Opie, Meghan Hockey, Dean Saunders, Courtney Bruscella, Felice Jacka, Megan Teychenne, Simon Rosenbaum, Khyati Banker, Sophie Mahoney, Monica Tembo, Jerry Lai, Niamh Mundell, Grace McKeon, Murat Yucel, Jane Speight, Pilvikki Absetz, Vincent Versace, Mary Lou Chatterton, Michael Berk, Sam Manger, Mohammadreza Mohebbi, Mark Morgan, Anna Chapman, Craig Bennett, Melissa O’Shea, Tetyana Rocks, Sarah Leach, Adrienne O’Neil

**Affiliations:** 1 Deakin University, IMPACT - the Institute for Mental and Physical Health and Clinical Translation, Food & Mood Centre, School of Medicine, Barwon Health, Geelong, Australia; 2 Deakin University, Geelong, Australia; 3 Barwon Health, Geelong, Australia; 4 University of New South Wales, Sydney, Australia; 5 Intersect Australia, Sydney, Australia; 6 Monash University, Melbourne, Australia; 7 Diabetes Victoria, Melbourne, Australia; 8 Tampere University, Tampere, Finland; 9 James Cook University, Townsville, Australia; 10 Bond University, Gold Coast, Australia; 11 GMHBA Health Insurance, Geelong, Australia

**Keywords:** Diet, Nutrition, Exercise, Physical activity, Depression, Mental health, Psychotherapy, Psychiatry, Mental disorders

## Abstract

**Background:**

There is increasing recognition of the substantial burden of mental health disorders at an individual and population level, including consequent demand on mental health services. Lifestyle-based mental healthcare offers an additional approach to existing services with potential to help alleviate system burden. Despite the latest Royal Australian New Zealand College of Psychiatrists guidelines recommending that lifestyle is a ‘first-line’, ‘non-negotiable’ treatment for mood disorders, few such programs exist within clinical practice. Additionally, there are limited data to determine whether lifestyle approaches are equivalent to established treatments. Using an individually randomised group treatment design, we aim to address this gap by evaluating an integrated lifestyle program (CALM) compared to an established therapy (psychotherapy), both delivered via telehealth. It is hypothesised that the CALM program will not be inferior to psychotherapy with respect to depressive symptoms at 8 weeks.

**Methods:**

The study is being conducted in partnership with Barwon Health’s Mental Health, Drugs & Alcohol Service (Geelong, Victoria), from which 184 participants from its service and surrounding regions are being recruited. Eligible participants with elevated psychological distress are being randomised to CALM or psychotherapy. Each takes a trans-diagnostic approach, and comprises four weekly (weeks 1-4) and two fortnightly (weeks 6 and 8) 90-min, group-based sessions delivered via Zoom (digital video conferencing platform). CALM focuses on enhancing knowledge, behavioural skills and support for improving dietary and physical activity behaviours, delivered by an Accredited Exercise Physiologist and Accredited Practising Dietitian. Psychotherapy uses cognitive behavioural therapy (CBT) delivered by a Psychologist or Clinical Psychologist, and Provisional Psychologist. Data collection occurs at baseline and 8 weeks. The primary outcome is depressive symptoms (assessed via the Patient Health Questionnaire-9) at 8 weeks. Societal and healthcare costs will be estimated to determine the cost-effectiveness of the CALM program. A process evaluation will determine its reach, adoption, implementation and maintenance.

**Discussion:**

If the CALM program is non-inferior to psychotherapy, this study will provide the first evidence to support lifestyle-based mental healthcare as an additional care model to support individuals experiencing psychological distress.

**Trial registration:**

Australia and New Zealand Clinical Trials Register (ANZCTR): ACTRN12621000387820, Registered 8 April 2021.

## Background

The burden of common mental disorders continues to grow with substantial impacts on health and major social and economic consquences globally [1]. While psychological interventions have been an effective treatment for mild-to-moderate depression and have shown comparable outcomes to pharmacotherapy [2], the increased demand for mental healthcare has caused lengthy waitlists for individuals seeking care for psychological distress [3]. For individuals in rural or remote communities, access to care is even more limited. Moreover, a proportion of individuals do not achieve full remission of symptoms from psychotherapy [4] or have a desire to seek other treatment options [5]. There is an urgent need for alternative approaches to established therapy which may alleviate the burden on mental health services, including responsive and flexible delivery models that reach those experiencing barriers to accessing conventional care.

Efficacy data supporting the use of lifestyle therapies as an adjunctive treatment for mental disorders are consistent and increasingly compelling [6]. A 12-week whole-of-diet intervention in 67 participants with moderate-to-severe depression was efficacious for reducing clinical depression [7]. It also found a cost saving of approximately $2600 per participant in the dietary condition due to reduced healthcare costs and reduced absenteeism [8]. A group-based study taking a similar approach to depression treatment yielded comparable findings and was also shown to be highly cost-effective [9]. These studies—backed up by level 1 evidence for dietary support to reduce depressive symptoms for a range of health conditions [10]—point to the potential of lifestyle therapies for producing large health benefits and cost savings in people with mental illness. Meta-analyses show physical activity interventions are also efficacious in treating depression [11, 12]. Literature reviews and preliminary experimental work indicate that physical activity can provide positive effects on neural structures [13], mental health [12, 14] and cognitive function [15]. These benefits also appear in the absence of clinical disorders, with a recent study reporting that an 8-week resistance exercise program significantly reduced anxiety symptoms in young adults without generalised anxiety disorder [16].

Telephone or web-based delivery is accepted increasingly as a feasible and effective delivery method for lifestyle programs [17]. ‘Telehealth’ refers to healthcare delivered via communications technologies rather than ‘in person’. Additionally, in the context of restricted physical mobility and pre-existing mental health concerns (such as agoraphobia and social anxiety), telehealth programs are highly applicable. A multimodal, integrated lifestyle telehealth intervention showed considerable promise for reducing depressive symptoms (assessed using the Patient Health Questionnaire 9 (PHQ-9)) compared to standard care, especially for those with a history of depression after a heart attack [18]. In addition, home exercise prescribed using a web-based exercise program resulted in higher confidence and engagement in physical activity compared to standard care [19]. As of 2021, the Australian Government identified telehealth delivery of mental health interventions as a key model of care [20].

The COVID-19 pandemic has led to further deteriorations in mental health and applied strain to mental health services. Compared to before the pandemic, the COVID-19 Mental Disorders Collaborators estimate that globally there has been a 27.6% increase in major depressive disorder cases and a 25.6% increase in anxiety disorders [21]. In addition to the direct impact of the disease itself (i.e. infection, fear of infection, loss of loved ones), the indirect impact of public health policy to promote infection control (such as lockdowns and restricted freedoms) may have exacerbated mental health symptoms in those with pre-existing mental health concerns [22]. Physical isolation may induce boredom, loneliness, frustration, anger, post-traumatic stress, financial loss, and stigma [23]—even in those with no history of mental illness. The deleterious effects of loneliness on health is equivalent to that of smoking [24]. It has been shown that, people living in the Australian state of Victoria, who have undergone the longest period of ‘lockdown’ anywhere in the world, have increased odds of experiencing clinically significant symptoms of depression and anxiety compared to those living in other areas of the country with less extensive lockdowns and restrictions [25]. This cohort also had significantly decreased odds of having high optimism about the future [25].

Despite the Royal Australian New Zealand College of Psychiatrists guidelines explicitly recommending that lifestyle approaches be a ‘first-line’, ‘non-negotiable’ treatment for mood disorders [26], there are surprisingly few studies from real world settings to guide implementation, nor to demonstrate their effects relative to an established psychotherapy. This paper presents the study protocol for the CALM (Curbing Anxiety and depression using Lifestyle Medicine) randomised controlled trial based in Victoria, Australia. The aim is to examine the relative effectiveness and cost-effectiveness of an integrated lifestyle program (CALM) compared to an established psychotherapy program—both delivered via telehealth. We hypothesise that the CALM program will not be inferior to the psychotherapy program for depressive symptoms (PHQ-9 scores) at 8 weeks.

## Methods

### Study design

This is a two-arm, parallel-group, individually randomised group treatment (IRGT), non-inferiority trial. This single-site study is currently recruiting *N* = 184 participants from the Barwon and surrounding regions (Victoria, Australia). Participants are individually randomised (1:1 allocation) to either of the two group-based programs, both delivered via telehealth. All participants complete assessments prior to randomisation (baseline) and at program completion (8 weeks). Recruitment and program delivery are anticipated to occur over 50 weeks, from May 2021 to April 2022.

### Study aims

The primary aim is to examine the effectiveness of the CALM program for reducing depressive symptoms compared to a standard psychotherapy program. The primary outcome will be change in depressive symptoms at 8 weeks as measured by the Patient Health Questionnaire-9 (PHQ-9) [27]. Secondary aims include: (i) examination of the efficacy of CALM versus psychotherapy on other health outcomes (anxiety symptoms, psychological distress, health behaviours, health functioning and cardiovascular disease biomarkers), (ii) investigation of the cost-effectiveness of the CALM program relative to psychotherapy from healthcare and societal perspectives, and (iii) a process evaluation by which to determine the reach, adoption, implementation and maintenance (RE-AIM) [28] of the CALM program beyond the research period.

### Participant eligibility

Participants are included if they are adults (aged 18 years or older), have capacity to provide informed consent, can converse in English, are willing to commit to six 90-min sessions over an 8-week period, have basic computer and internet literacy (able to access Zoom calls), and have a Distress Questionnaire-5 (DQ5) [29] score > 8 at enrolment. Participants are excluded if they have a known or suspected clinically unstable systemic medical disorder; severe food allergy, intolerance, aversions or malabsorption issue; a current or lapse/relapse of an eating disorder; or any other socio-cultural, religious, medical reasons which precludes their participation in a lifestyle intervention. During this trial period, participants cannot be involved in another intervention study. Participants cannot be pregnant, breastfeeding, or planning pregnancy within the next year. Participants are asked to provide blood and stool samples on two occasions (pre- and post-intervention). As this study aims to determine the effectiveness of the CALM intervention as an adjunctive therapy, ongoing pharmacological or other treatments during the intervention period is not an exclusion criterion. However, participants are excluded if they commence a new, duplicating treatment (e.g., psychotherapy) for a mental illness within a 1-month period prior to baseline or are experiencing an exacerbation of symptoms not adequately controlled by medication. Finally, participants must be deemed suitable for participation in a structured lifestyle or psychotherapy program for 8 weeks (i.e. not in crisis or suicidal at time of enrolment). Where evidence of acute suicidality or mental health crisis is identified during enrolment, a risk screening is conducted by a mental health clinician (TJ) to determine eligibility for participation. Individuals identified as in crisis or suicidal are directed to specialty services. Participants without access to a device and/or stable internet are loaned a device with mobile data.

### Recruitment and informed consent

The primary recruitment strategy is through Continuing Care Services (CCS) within the Barwon Health Mental Health, Drugs & Alcohol Service (MHDAS) in Geelong, Victoria, Australia. CCS provide medium-to-long term mental health services in community settings for consumers of all ages across the regional setting of Geelong and the surrounding regions (South West Victoria). Based on Australian remoteness classifications, this region covers Metropolitan Areas, Regional Centers, Large Rural Towns, Medium Rural Towns, and Small Rural Towns. A Referral Coordinator employed and based at MHDAS identifies potentially eligible participants from patient databases and case files and sends them an invitation to participate. This letter contains information about the study requirements and invites them to contact a member of the Deakin research team if they are interested in participating. To avoid potential coercion, the research team does not contact potential participants from MHDAS unless they have expressed interest.

Recruitment also occurs through community-based advertising such as flyers displayed around Deakin University campuses, Barwon Health sites, community hubs and General Practice clinics, media releases, newspaper articles, radio interviews, community talks and social media. Mental health services (e.g. Headspace) supports recruitment by providing flyers to clients at entry point and/or at assessment, with the objective of informing them of the trial and encouraging them to consider participating whilst on waiting list for headspace services and/or as an adjunct to headspace services.

Interested individuals undergo a brief eligibility check (including the DQ5, [29]) conducted over the phone with a study coordinator. If at any time they are identified as not meeting the inclusion criteria, they are informed that they are not eligible for inclusion in the trial but are provided with information on where to seek support. Eligible participants are invited to enrol in the study. Once e-consent is obtained, baseline assessments are booked at a time convenient to the participant.

Due to the individually randomised group treatment design, baseline assessment of eligible participants are ‘batched’ to ensure 50/50 randomisation to each arm whilst reducing the amount of time between baseline assessments and commencement of the program to which they have been assigned. In other words, baseline assessments are booked only once an adequate number of participants have enrolled to form groups (minimum 5, maximum 10, per group). At this point, participants are asked whether they have had any changes to medications or medical events since enrolment. If the time from enrolment to time of baseline assessments exceeds 2 weeks, they are re-assessed on the DQ5 to re-confirm eligibility.

### Data collection

To minimise interruptions caused by COVID-19 lockdowns, data collection is designed to involve minimal face-to-face contact. Demographic data (including age, sex, ethnicity, postcode, medications, nominated General Practitioner or case manager) are obtained via phone interview upon enrolment. At both baseline and 8-week follow-up, the blinded research assistant administers a battery of questionnaires via a Computer-Assisted Telephone Interview (CATI). This CATI takes approximately 30-60 min to complete and is conducted by a research assistant with specialised training. In addition to the CATI, the Mini-International Neuropsychiatric Interview (MINI) is administered in a separate Zoom call by a trained Clinical Psychologist or Provisional Psychologist. The MINI is a structured diagnostic interview designed to assess 17 of the most common mental health disorders [30]. It is used here to examine the prevalence of current and past major psychiatric disorders in the trial population.

In the absence of any COVID-19 related lockdown, consenting participants are also invited to complete an in-person visit at Australian Clinical Labs Collection Centre and Health Education and Research Building (both in Geelong). This visit cannot be administered via telephone. It includes a fasting blood test; weight, height, waist and hip measurements; blood pressure assessments; and muscular strength tests. Stool samples are provided by participants and returned via the post and stored in laboratory facilities at the Research Centre. As research assistants conducting data collection are blinded to participants’ program allocation, prior to the 8-week follow-up assessments participants are reminded not to reveal the group they have been assigned to in order for research assistants to retain blinded status.

If a participant chooses to withdraw from the study or is withdrawn by the research team (due to an adverse event, violating inclusion criteria, or if continuing would be detrimental to their wellbeing), data collected up until that time point will be used in the analysis of results, unless otherwise requested by the participant. A research assistant will also make a reasonable attempt to collect the primary outcome from withdrawing participants by organising a follow-up CATI.

#### Outcome measures

A summary of outcome measures and variables are displayed in Table 1. Schedule of enrolment, interventions and assessments are displayed in Table 2. The 9-item Patient Health Questionnaire-9 (PHQ-9) is the primary outcome of the study and is used to measure depressive symptoms. The PHQ-9 is based on the nine DSM-IV criteria for major depressive episode and is a frequently used clinical and research as a measure of depressive symptoms [27]. It assesses the proportion of days in the past 2 weeks that the respondent experienced various depressive symptoms on a 4-point scale from “Not at all” (0) to “Nearly every day” [3]. Scores range from 0 to 27. A cut-point of > 10 has been identified as providing an important threshold for identifying Diagnostic and Statistical Manual of Mental Disorders (DSM-IV) congruent major depression and shows good sensitivity and specificity [27] (Note: the current version DSM-V has consistent diagnostic criteria to DSM-IV). Of note, a different outcome measure was used to select the sample (i.e., the DQ5) to reduce regression to the mean, which can inflate effect sizes [31].

**Table 1:** Summary of assessments

Primary Outcome	Assessment
Depressive symptoms	9-item Patient Health Questionnaire-9 (PHQ-9) [27]
Secondary Outcomes	
Anxiety symptoms	7-item Generalised Anxiety Disorder scale (GAD-7) [32]
Anxiety concerning the COVID-19 pandemic	5-item Coronavirus Anxiety Scale (CAS) [33]
Non-specific psychological distress*	10-item Kessler-10 (K-10) [34]
Perceived social support	4-item (abbreviated) Medical Outcome Study Social Support Survey (MOS-SSS) [35]
Substance use	8-item Alcohol, Smoking and Substance Involvement Screening Test (ASSIST) [36]
Sleep hygiene	7-item Insomnia Severity Index (ISI) [37]
Health-related quality of life	12-item Assessment of Quality of Life (AQoL 4D) [38]
Health service use	Self reported use of prescription and over the counter medications, health professional visits, hospitalisations, absenteeism and presenteeism over the past 8 weeks
Stool consistency	4-item (modified) Bristol Stool Form Scale (BSFS) [39]
Psychosis symptoms	7-item Early Psychosis Questionnaire [40]
Physical activity levels	5-item Simple Physical Activity Questionnaire (SIMPAQ) [41, 42] and a modified Borg scale [43]
IBS diagnosis	1-item self-developed question asks participants if they have been told they have irritable bowel syndrome (IBS) by a general practitioner or gastroenterologist
High-density lipoprotein, Lower-density lipoprotein, total cholesterol	Fasting blood samples
Triglycerides, blood glucose	Fasting blood samples
Cardiovascular health	Systolic and diastolic blood pressure measured using an automatic sphygmomanometer
Waist circumference, hip circumference, height, weight	Height (to the nearest 0.1 cm), body weight (to the nearest 0.1 kg), waist circumference (to the nearest 0.1 cm) and hip circumference (to the nearest 0.1 cm)
Lower body muscular strength and upper body muscular strength	30-s sit-to-stand and 30-s bicep curl test
Diet intake	Dietary Questionnaire for Epidemiological Studies version 3.2 (DQES v3.2)—a modified version of the food frequency questionnaire developed by Cancer Council Victoria [44]
Gut microbiome composition	Stool collection (OMNIgene kits) [45]
Effect modifiers	
Health-related social needs	15-item American Academy of Family Physicians’ Social Needs Screening Tool [46]
Medication adherence	8-item Morisky Medication Adherence Scale (MMAS) [47]
Perceived self-efficacy	6-item (abbreviated) General Self-Efficacy Scale (GSE) [48]
Readiness to make changes in behaviour prior to the intervention [49]	3-item Readiness to Change Questionnaire (RCQ)
Treatment expectancy and rationale credibility	6-item Credibility/Expectancy Questionnaire (CEQ) [50]
Prevalence of current and past major psychiatric disorder	Mini-International Neuropsychiatric Interview (MINI) [30]

*K-10 is also used as a safety measure

**Table 2:**
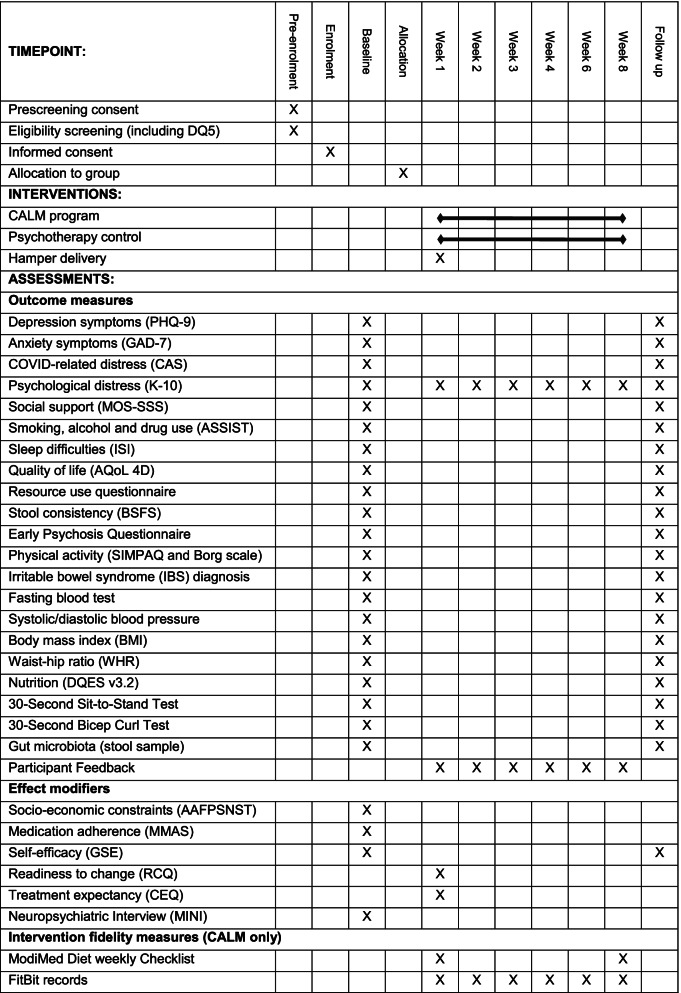
Schedule of enrolment, interventions and assessments

The secondary outcome measures were chosen as they assess various relevant aspects of mood including stress, anxiety, depression and health-related quality of life. These questionnaires assessed mood across various time periods (past week to past month). Administration via CATI was chosen to reduce burden on participants. They are also given a paper copy of the questionnaires to follow along. If too burdensome, participants have the option of completing the CATI over two separate calls, or alternatively complete as much as they can. The CATI is structured in order of importance of outcome measures (primary outcome first) to reduce missing data for the primary outcome and ensure retention of participants.

#### Safety

Procedural safeguards are in place for participants exhibiting deterioration in mental health, such as signs of suicidality, sub-optimal medication taking, an adverse event or elevation in psychological distress. Participants complete a fortnightly adverse events questionnaire, which is overseen by the study coordinator and medical personnel. In addition to being administered pre- and post-intervention, the K-10 is also administered each week during the intervention period to assess participant’s levels of generalised psychological distress. The K-10 consists of 10 items to measure non-specific psychological distress [34] and is commonly used in clinical and epidemiological contexts. Scores range from 10 to 50, with higher scores indicating more severe psychological distress. The K-10 is used to examine whether the interventions are having a positive effect on psychological distress throughout the course of treatment. An increase by more than 0.5 standard deviations, or a K10 score increase above 30, between sessions initiates safety protocol procedures.

An independent Data Safety Monitoring Board (DSMB) is overseeing the trial to maximise participant safety and identify patterns in adverse events that may be related to either program. The DSMB consists of members who collectively have experience in the lifestyle based interventions, digital interventions, biostatistics, and randomised clinical trials.

#### Intervention adherence and integrity

Participants’ attendance at each of the intervention sessions are recorded by the facilitators. Completion of the intervention is categorised as 50% or greater exposure/attendance (i.e. 3 or more sessions). At the conclusion of each telehealth session, the facilitators present five questions with Likert-style response options to participants to obtain session feedback. The results of these polls are used to guide the structure of future sessions to enhance participant engagement. All sessions are recorded on Zoom unless a participant does not consent to the recording. Approximately 10% of session recordings are assessed independently by two research staff to determine fidelity to both the CALM and psychotherapy conditions.

For participants in the CALM intervention arm only, the Mod*i*Med Diet Weekly Checklist [51] is used to assess how fully participants are engaging in a modified Mediterranean diet, as promoted by the CALM intervention. Engagement in the CALM physical activity objectives is monitored continually throughout the intervention period via the active minutes output obtained from FitBits that are provided to participants allocated to the CALM ‘lifestyle’ program at trial commencement. They are also asked to complete two items from the SIMPAQ and Borg scale to capture physical activity intensity on a weekly basis.

### Study conditions

All group-based telehealth sessions are delivered using the Zoom for Education platform; a secure, reliable, encrypted video conferencing facility. This platform allows multiple individuals to access a meeting via a computer, tablet or smartphone. Sessions are scheduled to be 90 min, with the option of a 15-min ‘drop-in’ before and after each session.

#### ‘CALM’ lifestyle program

The CALM lifestyle program has been developed by Accredited Practising Dietitians (APD) and Accredited Exercise Physiologists (EP), with the overarching goal to support positive lifestyle changes for mental health. CALM content is derived from the Mod*i*Med Diet used in the SMILES trial [7, 51], the Finnish Diabetes Prevention Study (DPS) [52], the GOAL Program [53], and the Australian Greater Green Triangle Diabetes Prevention Project (GGT DPP) [54]. These studies have all demonstrated successes in achieving improvements in physical and/or mental health outcomes, and hence were considered ideal models in which to develop CALM. The study goals were modified and refined from the original diabetes prevention programs based on the most-up-to date evidence in Lifestyle Psychiatry [6, 10, 55]. Thus, the goals of the CALM program are for participants to achieve:

1. No more than 10% of energy from saturated fats,
2. At least 15 g/1000 kcal fibre (approx. 30 – 45 g dietary fibre daily),
3. 150mins/week of moderate physical activity (or 75mins/week of vigorous physical activity – or equivalent combination of both).

Importantly, the CALM lifestyle program does not have a weight loss goal, which is based on evidence demonstrating that associations between diet quality and depression are independent of body weight (e.g., [7, 56]). Moreover, shifting the emphasis (i.e. from a weight-focussed to a health-focused paradigm) is consistent with the Health at Every Size® movement [57], and believed to help reduce the pressure to lose weight, which can add another mental health burden and challenge [58].

While the CALM program is evidence-based and manualised to allow for replication, it also harnesses and is guided by peer-to-peer interaction and participant discussion, and is tailored to the specific needs of the group (e.g., motivation levels, confidence, skills, preferred learning styles, knowledge, health literacy). At each session, participants in collaboration with facilitators, are encouraged to set relevant goals for achieving positive lifestyle change. While the program focus primarily relates to nutrition and physical activity, participants may nominate other lifestyle targets that are critical to mental health (e.g., alcohol, smoking, substance use, sleep hygiene). Facilitators aim to develop rapport and a comfortable, respectful, sensitive and non-judgmental environment. All facilitators are highly skilled, with advanced training in motivational interviewing, health coaching, goal setting and mindfulness.

Example content and activities include: key foods and nutrients for mental health, nutrition recommendations for mental health, physical activity recommendations for general health and mental health and safety considerations (adapted from [6, 59]), creating healthy convenient meals and snacks, shopping lists, practical physical activity examples, physical activity ‘snacks’ [60], label reading, barriers and enablers to lifestyle changes, mindful eating, mindful physical activity, recipe modification and support groups. Additionally, at the start of each session, participants are provided with an opportunity to ask questions arising from or since the previous session, discuss their goals and homework. At the final session, goals achieved during the intervention period are discussed and summarised. Further, discussions include ways to stay motivated, maintaining changes and dealing with set-backs, with longer-term strategies developed to support sustainable changes, including sources for additional information and peer support.

To encourage engagement with healthful dietary habits, participants are sent a food hamper at completion of their first session, which contains the key components of the Mod*i*Med diet to inspire and promote dietary change. This strategy has previously been used to successfully promote engagement in RCTs of other Mediterranean-type dietary approaches [7, 61]. For the physical activity sessions, participants are provided with a resistance band as a convenient, portable, and effective alternative to free weights and weight machines. They are also sent a FitBit Charge 2 to self-monitor their physical activity (pedometer). Participants are also encouraged to complete a Mod*i*Med checklist, a physical activity tracker and utilise health apps, to increase their engagement and build on skills learnt throughout the sessions.

#### Psychotherapy program

Cognitive Behavioural Therapy is widely recommended as a gold-standard psychological intervention for multiple mental health presentations [4, 62, 63], and has demonstrated effectiveness when delivered in groups (e.g., [64, 65]) and via telehealth (e.g., [66, 67]).

The psychotherapy program is a transdiagnostic CBT group adapted from the manualised Mood Management Course that was developed by the Centre for Clinical Interventions (CCI) [68]. The psychotherapy program also incorporates mindfulness practices, with a growing body of research suggesting that the integration of mindfulness and CBT can improve clinical outcomes [69, 70].

CBT proposes that the cognitive and behavioural factors which maintain mental health distress are amenable to change through recognising and challenging these unhelpful patterns [71]. As such, the psychotherapy group aims to develop skills in self-awareness, identifying and managing unhelpful thoughts and behaviours, and learning and practicing self-management strategies. Each session includes guided mindfulness practice. Each group is facilitated by a clinical or registered psychologist and a provisional psychologist.

To ensure parity with the CALM ‘lifestyle’ program, participants are sent a self-soothe hamper at commencement of the program, which includes items such as a colouring book, head massager, and stress ball. Participants are also encouraged to utilise mental health apps, to increase their engagement with skills such as mindfulness, breathing, thought logs, and cognitive disputation.

#### Randomization and blinding

Upon enrolment, participants are assigned a unique study identification (ID) number for randomisation purposes. After baseline assessment is complete, participants are assigned randomly to either the CALM group or the psychotherapy group. Allocation to the intervention arm is conducted using computer-generated block randomisation in a 1:1 ratio (CALM to psychotherapy). The allocation sequence is generated by an independent statistician. As randomisation occurs only when there are sufficient participants to form groups (minimum *n* = 5 per group), block randomisation is not feasible. Rather, a simple random allocation method is used within groups of participants which are ready to be randomised.

The Study Coordinator (unblinded) provides each participant with the schedule (for the group to which they have been allocated) and the technical support information (e.g., Zoom download, set up).

### Sample size

To assure non-inferiority hypothesis on the primary outcome, the maximum allowed upper limit of the 95% CIs (one-sided alpha of 5% was used for non-inferiority margin) of between-group mean difference on the PHQ-9 will be no larger than 2. This was based both on statistical reasoning (sample size and recruitment feasibility over the funded 18-month study period), clinical judgement and other psychotherapy trials using non-inferiority designs [72]. Assuming a standard deviation of 4, one-sided type I error = 0.025 and 80% power, a total sample size of *N* = 160 (*n* = 80 participants assigned to each group) will be required and inflated by 15% (*N* = 184) to allow for comparable attrition to that observed in our other telehealth trials (e.g., [18]).

### Data analyses

To compare continuous outcomes on the PHQ-9 in the primary analysis, between-group mean difference and confidence intervals (CIs) will be estimated using generalised estimating equation techniques, with Huber Sandwich Estimator of variance to account for clustering [31, 73]. One-sided type I error of 0.025 will be used for all non-inferiority analyses. Effect sizes will be calculated using Cohen’s d. Exploratory analyses non-equivalence comparisons will examine remission (post-treatment score < optimal cut-score for a probable diagnosis of depression on PHQ-9 in participants who initially scored above Threshold) and recovery rate (reduction of at least 50% of pre-treatment PHQ-9 scores) rates. Two-sided alpha of 5% will be used. Reliable improvement is defined as a reduction of > 5 points on PHQ-9 scores based on severity classifications pre-to-post treatment.

### Economic evaluation

A within-trial economic analysis will be conducted from health sector and partial societal perspectives by including intervention costs, the cost of other healthcare resources used by participants during the trial period, and lost productivity. Standard Australian unit costs (i.e. Pharmaceutical Benefits Schedule) will be applied and the average Australian wage rate plus 25% overhead costs used to value lost work time (the Human Capital Approach). Differences in total costs from health sector and partial societal perspectives will be compared with differences from multiple outcomes (quality-adjusted life years calculated from AQoL-4D utility values, PHQ-9, etc.) between groups.

### Data management

Participant details including medical history, medications and program session notes are entered into a Clinical Trial database, RealTime Software Solutions, LLC. Access to the RealTime database is password-protected, and study personnel are given individual user IDs and passwords. Baseline, weekly and 8-week, questionnaire data are collected through REDCap, stored securely at Deakin University’s Research Data Store on password protected servers. Zoom recordings are encrypted and stored locally on Deakin University’s password-protected servers. Stool samples are stored in a − 80 °C freezer housed in a secure, swipe-card protected facility at the Geelong Centre for Emerging & Infectious Diseases laboratory. Blood samples are analysed by Australian Clinical Labs and results uploaded to their online portal. This portal is password protected and only accessible to the research team.

### Ethics and dissemination

Approval to conduct the study was received from Human Research Ethics Committees of Barwon Health (20/199) and Deakin University (2021-166). The study is being conducted in accordance with the National Health and Medical Research Council (NHMRC) National Statement on Ethical Conduct in Human Research (2007) and the Note for Guidance on Good Clinical Practice (CPMP/ICH-135/95). Individual, electronic consent occurs prior to any testing procedures taking place. As both programs are designed to be adjunctive therapy, all participants are advised to continue usual care from their treating clinician (if relevant) while participating in the study. In accordance with the NHMRC Open Access Policy, research findings will be disseminated as widely as possible including: an open access repository, conferences proceedings and presentations and peer reviewed journals. Findings will be reported using the Consolidated Standards of Reporting Trials (CONSORT) statement [74].

## Discussion

Despite recent guideline recommendations that lifestyle approaches (i.e. healthful dietary and physical activity behaviours) be a ‘first-line’, ‘non-negotiable’ treatment for mood disorders, this approach is seldom delivered by a registered dietitian and/or exercise physiologist as part of mainstream clinical practice. The CALM program is a discrete service model that provides an opportunity for adults with (or at risk of developing) a mental disorder to access lifestyle-based mental health care. Lifestyle approaches have also been proven to address shared risk factors for comorbid non-communicable medical disorders [75]. In the context of a global pandemic when psychological distress is high, additional strain has been placed upon an already overburdened mental healthcare system and face-to-face care has been disrupted, the telehealth model employed as part of the CALM trial has the potential to provide an additional pathway to ease such barriers. If CALM is non-inferior to established psychotherapy, it has the potential not only to provide additional and supplementary care to individuals with mental health concerns but to enable the workforce of dietitians and exercise professionals to support mental healthcare during COVID-19 and beyond. For the first time, our study will provide real-world data on the effectiveness and cost-effectiveness of an integrated lifestyle program compared to established psychotherapy and is anticipated to produce dual mental and physical health benefits that may have long-term health and cost-savings.

## Data Availability

Not applicable.

## Abbreviations

ANZCTR: Australia and New Zealand Clinical Trials Register
PHQ-9: Patient Health Questionnaire 9
CALM: Curbing Anxiety and depression using Lifestyle Medicine
IRGT: individually randomised group treatment
RE-AIM: reach, adoption, implementation and maintenance
DQ5: Distress Questionnaire-5
MHDAS: Mental Health, Drugs & Alcohol Service
CCS: Continuing Care Services
CATI: Computer-Assisted Telephone Interview
MINI: Mini-International Neuropsychiatric Interview
DSM: Diagnostic and Statistical Manual of Mental Disorders
GAD-7: Generalised Anxiety Disorder scale
CAS: Coronavirus Anxiety Scale
K-10: Kessler-10
MOS-SSS: Medical Outcome Study Social Support Survey
ASSIST: Alcohol, Smoking and Substance Involvement Screening Test
ISI: Insomnia Severity Index
AQoL 4D: Assessment of Quality of Life
BSFS: Bristol Stool Form Scale;
SIMPAQ: Simple Physical Activity Questionnaire
IBS: irritable bowel syndrome
DQES v3.2: Dietary Questionnaire for Epidemiological Studies version 3.2
MMAS: Morisky Medication Adherence Scale
GSE: General Self-Efficacy Scale
RCQ: Readiness to Change Questionnaire
CEQ: Credibility/Expectancy Questionnaire
BMI: Body mass index
WHR: Waist-hip ratio
DSMB: Data Safety Monitoring Board
APD: Accredited Practising Dietitians
EP: Accredited Exercise Physiologists
DPS: Diabetes Prevention Study
GGT DPP: Greater Green Triangle Diabetes Prevention Project
CBT: Cognitive Behavioural Therapy
CCI: Centre for Clinical Interventions
CIs: Confidence Intervals
NHMRC: National Health and Medical Research Council
CONSORT: Consolidated Standards of Reporting Trials

## References

[1] Rehm J, Shield KD (2019). Global burden of disease and the impact of mental and addictive disorders. Curr Psychiatry Rep.

[2] Biesheuvel-Leliefeld KE, Kok GD, Bockting CL, Cuijpers P, Hollon SD, van Marwijk HW (2015). Effectiveness of psychological interventions in preventing recurrence of depressive disorder: meta-analysis and meta-regression. J Affect Disord.

[3] Ashcroft R, Donnelly C, Dancey M, Gill S, Lam S, Kourgiantakis T (2021). Primary care teams’ experiences of delivering mental health care during the COVID-19 pandemic: a qualitative study. BMC Fam Pract.

[4] Santoft F, Axelsson E, Öst LG, Hedman-Lagerlöf M, Fust J, Hedman-Lagerlöf E (2019). Cognitive behaviour therapy for depression in primary care: systematic review and meta-analysis. Psychol Med.

[5] Dunlop BW, Kelley ME, Aponte-Rivera V, Mletzko-Crowe T, Kinkead B, Ritchie JC (2017). Effects of patient preferences on outcomes in the predictors of remission in depression to individual and combined treatments (PReDICT) study. Am J Psychiatry.

[6] Firth J, Solmi M, Wootton RE, Vancampfort D, Schuch FB, Hoare E (2020). A meta-review of "lifestyle psychiatry": the role of exercise, smoking, diet and sleep in the prevention and treatment of mental disorders. World Psychiatry.

[7] Jacka FN, O’Neil A, Opie R, Itsiopoulos C, Cotton S, Mohebbi M (2017). A randomised controlled trial of dietary improvement for adults with major depression (the ‘SMILES’ trial). BMC Med.

[8] Chatterton ML, Mihalopoulos C, O’Neil A, Itsiopoulos C, Opie R, Castle D (2018). Economic evaluation of a dietary intervention for adults with major depression (the “SMILES” trial). BMC Public Health.

[9] Segal L, Twizeyemariya A, Zarnowiecki D, Niyonsenga T, Bogomolova S, Wilson A (2020). Cost effectiveness and cost-utility analysis of a group-based diet intervention for treating major depression - the HELFIMED trial. Nutr Neurosci.

[10] Firth J, Marx W, Dash S, Carney R, Teasdale SB, Solmi M (2019). The effects of dietary improvement on symptoms of depression and anxiety: a Meta-analysis of randomized controlled trials. Psychosom Med.

[11] Ashdown-Franks G, Firth J, Carney R, Carvalho AF, Hallgren M, Koyanagi A (2020). Exercise as medicine for mental and substance use disorders: a Meta-review of the benefits for neuropsychiatric and cognitive outcomes. Sports Med.

[12] Schuch FB, Vancampfort D, Richards J, Rosenbaum S, Ward PB, Stubbs B (2016). Exercise as a treatment for depression: a meta-analysis adjusting for publication bias. J Psychiatr Res.

[13] Firth J, Stubbs B, Vancampfort D, Schuch F, Lagopoulos J, Rosenbaum S (2018). Effect of aerobic exercise on hippocampal volume in humans: a systematic review and meta-analysis. Neuroimage..

[14] Teychenne M, White RL, Richards J, Schuch FB, Rosenbaum S, Bennie JA (2020). Do we need physical activity guidelines for mental health: what does the evidence tell us?. Ment Health Phys Act.

[15] Hendrikse J, Chye Y, Thompson S, Rogasch NC, Suo C, Coxon J, et al. The effects of regular aerobic exercise on hippocampal structure and function. bioRxiv. 2020:2020.08.14.250688. 10.1002/hipo.23397 34961996

[16] Gordon BR, McDowell CP, Lyons M, Herring MP (2020). Resistance exercise training for anxiety and worry symptoms among young adults: a randomized controlled trial. Sci Rep.

[17] Griffiths KM, Farrer L, Christensen H (2010). The efficacy of internet interventions for depression and anxiety disorders: a review of randomised controlled trials. Med J Aust.

[18] O’Neil A, Taylor B, Sanderson K, Cyril S, Chan B, Hawkes A, et al. Efficacy and feasibility of a tele-health intervention for acute coronary syndrome patients with depression: results of the “MoodCare” randomized controlled trial. Ann Behav Med. 2014;48. 10.1007/s12160-014-9592-0 24570217

[19] Bennell KL, Marshall CJ, Dobson F, Kasza J, Lonsdale C, Hinman RS (2019). Does a web-based exercise programming system improve home exercise adherence for people with musculoskeletal conditions?: a randomized controlled trial. Am J Phys Med Rehabil.

[20] Parliament of the Commonwealth of Australia. Mental health and suicide prevention - final report. In: Prevention HoRSCoMHaS, editor. Canberra2021.

[21] COVID-19 Mental Disorders Collaborators. Global prevalence and burden of depressive and anxiety disorders in 204 countries and territories in 2020 due to the COVID-19 pandemic. Lancet. 2021;398(10312):1700–12. 10.1016/S0140-6736(21)02143-7. 10.1016/S0140-6736(21)02143-7 PMC8500697 34634250

[22] O'Neil A, Nicholls SJ, Redfern J, Brown A, Hare DL (2020). Mental health and psychosocial challenges in the COVID-19 pandemic: food for thought for cardiovascular health care professionals. Heart Lung Circ.

[23] Brooks SK, Webster RK, Smith LE, Woodland L, Wessely S, Greenberg N (2020). The psychological impact of quarantine and how to reduce it: rapid review of the evidence. Lancet.

[24] Valtorta NK, Kanaan M, Gilbody S, Ronzi S, Hanratty B (2016). Loneliness and social isolation as risk factors for coronary heart disease and stroke: systematic review and meta-analysis of longitudinal observational studies. Heart..

[25] Fisher J, Tran T, Hammarberg K, Nguyen H, Stocker R, Rowe H (2021). Quantifying the mental health burden of the most severe covid-19 restrictions: a natural experiment. J Affect Disord.

[26] Malhi GS, Bassett D, Boyce P, Bryant R, Fitzgerald PB, Fritz K (2015). Royal Australian and New Zealand College of Psychiatrists clinical practice guidelines for mood disorders. Aust N Z J Psychiatry.

[27] Kroenke K, Spitzer RL, Williams JB (2001). The PHQ-9: validity of a brief depression severity measure. J Gen Intern Med.

[28] Glasgow RE, Vogt TM, Boles SM (1999). Evaluating the public health impact of health promotion interventions: the RE-AIM framework. Am J Public Health.

[29] Batterham PJ, Sunderland M, Carragher N, Calear AL, Mackinnon AJ, Slade T (2016). The Distress Questionnaire-5: population screener for psychological distress was more accurate than the K6/K10. J Clin Epidemiol.

[30] Sheehan DV, Lecrubier Y, Sheehan KH, Amorim P, Janavs J, Weiller E, et al. The Mini-International Neuropsychiatric Interview (M.I.N.I.): the development and validation of a structured diagnostic psychiatric interview for DSM-IV and ICD-10. J Clin Psychiatry. 1998;59 Suppl 20:22-33;quiz 4-57. 9881538

[31] Christensen H, Griffiths KM, Mackinnon AJ, Kalia K, Batterham PJ, Kenardy J, et al. Protocol for a randomised controlled trial investigating the effectiveness of an online e health application for the prevention of Generalised Anxiety Disorder. BMC Psychiatry. 2010;10:25-. 10.1186/1471-244X-10-25 PMC2848219 20302678

[32] Spitzer RL, Kroenke K, Williams JB, Löwe B (2006). A brief measure for assessing generalized anxiety disorder: the GAD-7. Arch Intern Med.

[33] Lee SA (2020). Coronavirus anxiety scale: a brief mental health screener for COVID-19 related anxiety. Death Stud.

[34] Andrews G, Slade T (2001). Interpreting scores on the Kessler psychological distress scale (K10). Aust N Z J Public Health.

[35] Gjesfjeld CD, Greeno CG, Kim KH (2008). A confirmatory factor analysis of an abbreviated social support instrument: the MOS-SSS. Res Soc Work Pract.

[36] Humeniuk R, Henry-Edwards S, Ali R, Poznyak V, Monteiro MG, World Health O. The alcohol, smoking and substance involvement screening test (ASSIST): manual for use in primary care / prepared by R. HumeniukƯ [et al]. Geneva: World Health Organization; 2010.

[37] Bastien CH, Vallières A, Morin CM (2001). Validation of the insomnia severity index as an outcome measure for insomnia research. Sleep Med.

[38] Richardson J, Iezzi A, Khan MA, Maxwell A (2014). Validity and reliability of the assessment of quality of life (AQoL)-8D multi-attribute utility instrument. Patient..

[39] Blake MR, Raker JM, Whelan K (2016). Validity and reliability of the Bristol stool form scale in healthy adults and patients with diarrhoea-predominant irritable bowel syndrome. Aliment Pharmacol Ther.

[40] headspace National Youth Mental Health Foundation. headspace Early Psychosis [Available from: https://headspace.org.au/assets/Uploads/11491-Early-Psychosis-GPChecklist-FA-HR-nocrops2.pdf.

[41] Rosenbaum S, Ward PB (2016). The simple physical activity questionnaire. Lancet Psychiatry.

[42] Rosenbaum S, Morell R, Abdel-Baki A, Ahmadpanah M, Anilkumar TV, Baie L (2020). Assessing physical activity in people with mental illness: 23-country reliability and validity of the simple physical activity questionnaire (SIMPAQ). BMC Psychiatry.

[43] Williams N (2017). The Borg rating of perceived exertion (RPE) scale. Occup Med.

[44] Hebden L, Kostan E, O'Leary F, Hodge A, Allman-Farinelli M. Validity and reproducibility of a food frequency questionnaire as a measure of recent dietary intake in young adults. PLoS ONE. 2013;8(9):e75156-e. 10.1371/journal.pone.0075156 PMC3776736 24058660

[45] DNA Genotek. Microbial collection and stabilization kits Collection from feces / stool samples [Available from: https://www.dnagenotek.com/row/products/collection-microbiome/omnigene-gut/OM-200.html.

[46] American Academy of Family Physicians. Social Needs Screening Tool 2018 [Available from: https://www.aafp.org/dam/AAFP/documents/patient_care/everyone_project/hops19-physician-form-sdoh.pdf.

[47] Muntner P, Joyce C, Holt E, He J, Morisky D, Webber LS (2011). Defining the minimal detectable change in scores on the eight-item Morisky medication adherence scale. Ann Pharmacother.

[48] Schwarzer R, Jerusalem M, Weinman J, Wright S, Johnston M (1995). Generalized self-efficacy scale. Measures in health psychology: a user’s portfolio.

[49] Prochaska JO, Norcross JC (2001). Stages of change. Psychother Theory Res Pract Train.

[50] Devilly GJ, Borkovec TD (2000). Psychometric properties of the credibility/expectancy questionnaire. J Behav Ther Exp Psychiatry.

[51] Opie RS, O'Neil A, Jacka FN, Pizzinga J, Itsiopoulos C (2018). A modified Mediterranean dietary intervention for adults with major depression: dietary protocol and feasibility data from the SMILES trial. Nutr Neurosci.

[52] Lindström J, Peltonen M, Eriksson JG, Ilanne-Parikka P, Aunola S, Keinänen-Kiukaanniemi S (2013). Improved lifestyle and decreased diabetes risk over 13 years: long-term follow-up of the randomised Finnish diabetes prevention study (DPS). Diabetologia..

[53] Absetz P, Oldenburg B, Hankonen N, Valve R, Heinonen H, Nissinen A (2009). Type 2 diabetes prevention in the real world: three-year results of the GOAL lifestyle implementation trial. Diabetes Care.

[54] Laatikainen T, Dunbar JA, Chapman A, Kilkkinen A, Vartiainen E, Heistaro S (2007). Prevention of type 2 diabetes by lifestyle intervention in an Australian primary health care setting: greater green triangle (GGT) diabetes prevention project. BMC Public Health.

[55] Xu Y, Zeng L, Zou K, Shan S, Wang X, Xiong J (2021). Role of dietary factors in the prevention and treatment for depression: an umbrella review of meta-analyses of prospective studies. Transl Psychiatry.

[56] Jacka FN, Pasco JA, Mykletun A, Williams LJ, Hodge AM, O'Reilly SL (2010). Association of Western and traditional diets with depression and anxiety in women. Am J Psychiatry.

[57] Association for Size Diversity and Health. 2006 [Available from: https://asdah.org/.

[58] Penney TL, Kirk SFL (2015). The health at every size paradigm and obesity: missing empirical evidence may help push the reframing obesity debate forward. Am J Public Health.

[59] Bull FC, Al-Ansari SS, Biddle S, Borodulin K, Buman MP, Cardon G (2020). World Health Organization 2020 guidelines on physical activity and sedentary behaviour. Br J Sports Med.

[60] Sanders JP, Biddle SJH, Gokal K, Sherar LB, Skrybant M, Parretti HM (2021). ‘Snacktivity™’ to increase physical activity: time to try something different?. Prev Med.

[61] Martínez-González M, Corella D, Salas-Salvadó J, Ros E, Covas M-I, Fiol M (2010). Cohort profile: design and methods of the PREDIMED study. Int J Epidemiol.

[62] Carpenter JK, Andrews LA, Witcraft SM, Powers MB, Smits JAJ, Hofmann SG (2018). Cognitive behavioral therapy for anxiety and related disorders: a meta-analysis of randomized placebo-controlled trials. Depress Anxiety.

[63] Cuijpers P, Noma H, Karyotaki E, Cipriani A, Furukawa TA (2019). Effectiveness and acceptability of cognitive behavior therapy delivery formats in adults with depression: a network meta-analysis. JAMA Psychiatry.

[64] Okumura Y, Ichikura K (2014). Efficacy and acceptability of group cognitive behavioral therapy for depression: a systematic review and meta-analysis. J Affect Disord.

[65] Petrocelli J (2002). Effectiveness of group cognitive-behavioral therapy for general symptomatology: a meta-analysis. The Journal for Specialists in Group Work.

[66] Berryhill MB, Halli-Tierney A, Culmer N, Williams N, Betancourt A, King M (2019). Videoconferencing psychological therapy and anxiety: a systematic review. Fam Pract.

[67] Sijbrandij M, Kunovski I, Cuijpers P (2016). Effectiveness of internet-delivered cognitive behavioral therapy for posttraumatic stress disorder: a systematic review and meta-analysis. Depress Anxiety..

[68] Nathan P, Smith L, Rees C, Correia H, Juniper U, Kingsep P (2004). Mood management course: a cognitive Behavioural group treatment Programme for anxiety disorders and depression.

[69] Goldberg SB, Tucker RP, Greene PA, Davidson RJ, Wampold BE, Kearney DJ (2018). Mindfulness-based interventions for psychiatric disorders: a systematic review and meta-analysis. Clin Psychol Rev.

[70] Segal ZV, Williams JMG, Teasdale JD (2013). Mindfulness-based cognitive therapy for depression.

[71] Beck JS (2011). Cognitive behavior therapy: basics and beyond.

[72] Titov N, Andrews G, Davies M, McIntyre K, Robinson E, Solley K. Internet treatment for depression: a randomized controlled trial comparing clinician vs. technician assistance. PLoS ONE. 2010;5(6):e10939. 10.1371/journal.pone.0010939 PMC2882336 20544030

[73] Huber PJ, Editor the behavior of maximum likelihood estimates under nonstandard conditions. Proceedings of the fifth Berkeley symposium on mathematical statistics and probability, volume 1: statistics; 1967 1967; Berkeley, Calif: University of California Press.

[74] Schulz KF, Altman DG, Moher D, the CG. CONSORT 2010 Statement: updated guidelines for reporting parallel group randomised trials. BMC Medicine. 2010;8(1):18. 10.1186/1741-7015-8-18 PMC2860339 20334633

[75] Dunbar JA, Jayawardena A, Johnson G, Roger K, Timoshanko A, Versace VL (2014). Scaling up diabetes prevention in Victoria, Australia: policy development, implementation, and evaluation. Diabetes Care.

